# A unique STK4 mutation truncating only the C-terminal SARAH domain results in a mild clinical phenotype despite severe T cell lymphopenia: Case report

**DOI:** 10.3389/fimmu.2024.1329610

**Published:** 2024-02-01

**Authors:** Bandar Al-Saud, Huda Alajlan, Hibah Alruwaili, Latifa Almoaibed, Amer Al-Mazrou, Hazem Ghebeh, Monther Al-Alwan, Anas M. Alazami

**Affiliations:** ^1^ Section of Pediatric Allergy/Immunology, Department of Pediatrics, King Faisal Specialist Hospital and Research Center, Riyadh, Saudi Arabia; ^2^ College of Medicine, Alfaisal University, Riyadh, Saudi Arabia; ^3^ Translational Genomics, Centre for Genomic Medicine, King Faisal Specialist Hospital and Research Centre, Riyadh, Saudi Arabia; ^4^ Cell Therapy and Immunobiology Department, King Faisal Specialist Hospital and Research Centre, Riyadh, Saudi Arabia

**Keywords:** primary immunodeficiency, lymphopenia, NGS, SARAH domain, case report

## Abstract

Mutations in STK4 (MST1) are implicated in a form of autosomal recessive combined immunodeficiency, resulting in recurrent infections (especially Epstein-Barr virus viremia), autoimmunity, and cardiac malformations. Here we report a patient with an atypically mild presentation of this disease, initially presenting with severe T cell lymphopenia (< 500 per mm^3^) and intermittent neutropenia, but now surviving well on immunoglobulins and prophylactic antibacterial treatment. She harbors a unique STK4 mutation that lies further downstream than all others reported to date. Unlike other published cases, her mRNA transcript is not vulnerable to nonsense mediated decay (NMD) and yields a truncated protein that is expected to lose only the C-terminal SARAH domain. This domain is critical for autodimerization and autophosphorylation. While exhibiting significant differences from controls, this patient’s T cell proliferation defects and susceptibility to apoptosis are not as severe as reported elsewhere. Expression of PD-1 is in line with healthy controls. Similarly, the dysregulation seen in immunophenotyping is not as pronounced as in other published cases. The nature of this mutation, enabling its evasion from NMD, provides a rare glimpse into the clinical and cellular features associated with the absence of a “null” phenotype of this protein.

## Introduction

First identified in *Drosophila*, the Hippo pathway contains genes with close orthologues in mammals which are critical for organ development and growth (1). Serine-threonine kinase 4 (STK4), also known as mammalian sterile 20-like 1 (MST1), is the orthologue of *Drosophila* Hpo and is an essential kinase within the canonical and non-canonical Hippo signaling pathways. It promotes an assortment of immune cell functions including B cell immunity (2) and T cell expansion and migration (3, 4). Its downstream induction of FOXO (forkhead box protein) family members such as FOXO1 and FOXO3 allows STK4 to orchestrate effective CD8 T cell responses to persistent viral infections, the formation of regulatory T cells, and overall T cell homeostasis (5).

Bi-allelic loss-of-function mutations in STK4 cause combined immunodeficiency (CID) (6, 7). Deficiency of this protein has been linked to recurrent infections of bacteria, fungi, and viruses, with nearly half of the patients exhibiting Epstein-Barr Virus (EBV) viremia and EBV-associated lymphoproliferative disorder (8), exacerbated by the significantly impaired response of interferon types I, II, and III (9). Malignancies are often reported due to the subsequent development of B cell lymphomas. Sporadic neutropenia, T and B cell lymphopenia, and an elevated risk of autoimmunity are common findings (6, 7). Additional documented features include short stature, primary cardiac T cell lymphoma, and a Castleman-like disorder (9–11). Immunological characterization studies show that with time, memory B cell and naïve (CD45RA+) T cell numbers drop drastically, and peripheral T cell survival is severely compromised along with impaired response to antigens (7).

In addition to its kinase activity, STK4 protein contains a Salvador/Rassf/Hippo (SARAH) domain at its C-terminal end which is required for dimerization (12). Here we report a patient with profound T cell lymphopenia but otherwise mild clinical presentation. Her novel truncating mutation in STK4, situated further downstream than any previously described, generates an RNA transcript that is resistant to NMD and therefore illustrates the human phenotype that is associated with the loss of only the SARAH domain on the STK4 protein.

## Case description

The patient is a 10-year-old female born to consanguineous parents from Saudi Arabia. The parents are asymptomatic and have a healthy 5-year-old son. The mother has a history of two first-trimester abortions for unknown reasons. At 4 years of age, the patient began having recurrent infections (accounting for three otitis media per year requiring antibiotics), plus urinary tract infections and recurrent oral thrush. Subsequently, she was admitted at the age of 7 years with severe gastroenteritis and her laboratory work up revealed leukopenia and normal immunoglobulin. She also experienced recurrent episodes of scalp hair loss and oral ulcers suspected to be secondary to nutritional deficit, which was managed by a course of omega-3 and zinc for 4 months with mild improvement. She also reported symptoms of recurrent frontal headache with photophobia and phonophobia (imaging was not done). The headache was relieved by rest and paracetamol, and was attributed to a family history of migraines. The patient had erythematous skin rash mainly on the trunk, sparing the face, and responded well to topical steroids.

At the age of 8 years, the patient was referred to us to investigate the possibility of primary immunodeficiency. At the time, physical assessment revealed weight and height within the 25^th^ percentile. Chest examination revealed good bilateral air entry and no added sound, heart sound was normal and the remainder of the exam was unremarkable. Her initial laboratory results (**Table 1**) showed neutropenia, lymphopenia, and high IgM. Her T cell counts were severely depressed, being in the atypical or leaky SCID range. B cell count was also low, but with normal NK and acceptable response to tetanus vaccines.

**Table 1:** Initial immunological and hematological laboratory results.

Variable	Patient ^a^	Normal range
WBC X 10^9^/L	1.7	4.3 – 11.3
HB g/L	124	110 – 150
MCV fL	81.4	75 - 95
Platelet X 10^9^/L	320	155 - 435
Neutrophils X 10^9^/L	2.8	1.35 – 7.5
Lymphocytes X 10^9^/L	1.04	1.9 – 4.9
Eosinophils X 10^9^/L	0.18	0.03 – 1
IgG g/L	10.9	5.4 – 13.6
IgA g/L	2.31	0.5 – 3.05
IgM g/L	6.42	0.31 – 2.08
IgE KU/L	34.5	5 - 500
CD 3/mm^3^	480	1700 - 1900
CD 4/mm^3^	389	800 – 1700
CD 8/mm^3^	72	700 - 1000
CD 19/mm^3^	148	400 - 800
CD16/56/mm^3^	161	200 - 400

a Sampled at the age of 8 years old.

She was evaluated by rheumatology for her history of recurrent hair loss, skin rash, and oral ulcers to rule out autoimmune diseases. Her initial autoimmune work up indicated normal ESR, negative for checked antibodies (anti-nuclear, anti-aPS aPT IgG/IgM, anti-endomyseal antibodies), normal anti tissue transglutaminase, anti-gliadin AB < 0.4, anti-cardiolipin IgA and IgG < 09.4, and normal C3 and C4 levels. These findings were not suggestive of any autoimmune disease. Echocardiography showed no cardiac anomalies.

She received all her vaccinations up to school age. There was no history of delayed milestones and she currently attends regular schooling.

The patient responded well to prophylactic antibiotic, antifungal, and intravenous immunoglobulin (IVIG) (0.4g/kg) every 4 weeks. She only had three admissions over the last 3 years with gastroenteritis; two were due to Clostridium difficile toxin, as stool samples were positive for toxigenic C. difficile by rapid DNA testing. The stool culture and stool for ova and parasites were otherwise negative, and the patient responded well to antibiotics.

## Results

### Identification of a novel STK4 mutation

**Figure 1:**
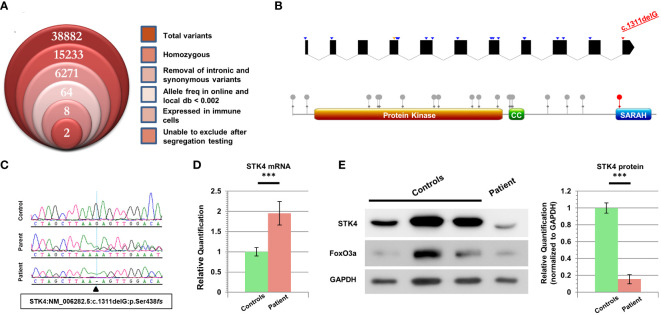
Characterization of cells harboring a novel *STK4* mutation. **(A)** Stacked Venn diagram indicating the total number of variants highlighted by WES, following the inclusion of each filter. **(B)** Top: schematic of the *STK4* gene illustrating the positions of previously reported mutations. Blue arrowheads denote truncating mutations, orange denotes missense, and our mutation is indicated in red. For the sake of clarity, UTR regions are not shown, and intronic regions are not drawn to scale (although coding portions of exons are to scale). The list of mutations is based on HGMD Professional (v 2023.1). Bottom: schematic of the STK4 protein highlighting the locations of domains and reported coding mutations. Our mutation is the only one whose impact is limited to loss of the C-terminal SARAH domain. **(C)** Sequence chromatograms of the patient, one parent, and a healthy control for comparison. **(D)** Real-time RT-PCR data for STK4 expression levels in the patient’s lymphoblastoid cells, versus combined data from three healthy controls. **(E)** Left: immunoblotting reveals low levels of truncated STK4 protein in the patient’s lymphoblastoid cells, as well as low levels of the downstream target FoxO3a. GAPDH serves as a loading control. Right: quantification of three independent immunoblots, utilizing ImageJ analysis, reveals that STK4 protein expression is significantly depressed in the patient. ***p < 0.001; unpaired Student’s *t*-test.

We recruited this family under an IRB-approved informed consent (see **Supplementary Materials**). Our study encompassed the patient and her parents, but her unaffected sibling was not available for recruitment. Genomic DNA from the patient was analyzed through whole exome sequencing, then filtered under the assumption of an autosomal recessive disorder due to the high consanguinity rates in the region. Additional parameters were employed as shown in **Figure 1A**. A total of eight variants were then highlighted for segregation testing. Some variants could not be sequenced due to the repetitive nature of the locus, but given the lack of heterozygosity in the parents they were dismissed. Only two variants clearly segregated with the disease state, but one of these (*CEP295*:NM_033395.2:p.E2318K) was in a gene linked to primary microcephaly and Bardet-Biedl syndrome, and hence incompatible with our patient’s presentation. The most promising variant was therefore a truncating mutation in the *STK4* gene (NM_006282.5:c.1311delG:p.S438*fs*), which was confirmed to be homozygous in the patient and heterozygous in her parents (**Figure 1C**).

Intriguingly, this frameshift mutation occurs in the last exon of *STK4*, which is more downstream than any of the other mutations reported for this gene to date (**Figure 1B**). The mutation is predicted to cause alteration and/or loss of the last 50 amino acid residues, which for a protein of this size (487 aa) constitutes just over 10%, and is expected to remove only the C-terminal SARAH domain, which participates in STK4 homo- as well as heterodimerization with other proteins (13). RT-PCR data revealed that transcripts of this gene were present in patient cells at nearly twice the quantity of our controls (**Figure 1D**) (details in **Supplementary Materials**). This significant difference suggested that the location of this variant in the last exon, just downstream of the final intron-exon boundary, was allowing the mutant transcript to escape NMD surveillance.

Immunoblotting of lysates from lymphoblastoid cells revealed the presence of the truncated form of STK4 in the patient. Contrary to the increased mRNA expression of this gene, the level of STK4 protein in the patient was significantly reduced (**Figure 1E**). FoxO3a, a direct downstream target of STK4 whose stability is dependent on STK4 phosphorylation, also showed low expression levels, and was at the lower range of what was observed in controls.

### Flow cytometry analysis of patient T cells

**Figure 2:**
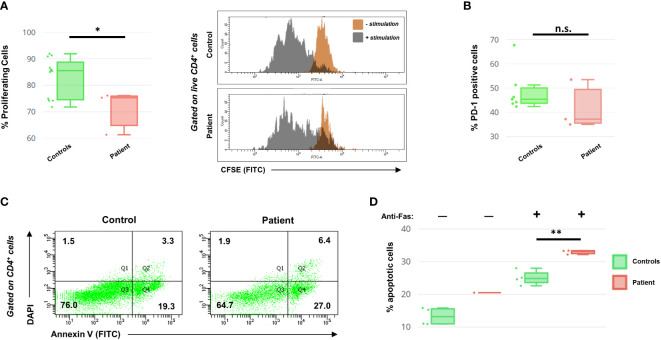
**(A)** Left: Box-and-whiskers summary of CFSE proliferation data on PBMCs from controls and the patient following 3 days of CD3/CD28 stimulation and gated on live (DAPI-) CD4+ T helper cells. Right: representative histograms of CFSE readout for CD4+ cells, in the presence or absence of stimulation. **(B)** PD-1 cell surface expression data, following 48 hours of CD3/CD28 stimulation. Data are presented as a percentage of live CD4+ cells. **(C)** Representative histograms of the DAPI/Annexin V cellular profile for patient and control CD4+ cells, following T cell activation with anti-Fas antibody challenge. The quadrants relate to necrosis (Q1), late apoptosis (Q2), healthy (Q3), and early apoptosis (Q4). Numbers indicate cell percentages within each quadrant. **(D)** Summary of the AICD data for activated control and patient CD4+ cells, following 72 hours of CD3/CD28 stimulation with or without 24 hours of anti-Fas incubation. Percentage shows the sum of both early and late apoptotic cells. Annexin V profiling of patient T cells in the absence of anti-Fas was conducted only once due to cell number limitations. Asterisks indicate significance levels (*p < 0.05, **p < 0.01; unpaired Student’s *t-*test). Error bars for **(A, B, D)** indicate SEMs. n.s., not significant.

Because of the role STK4 plays in modulating T cell health and activity, we subjected patient PBMCs to CFSE, a reagent that allows fluorescent staining of cells to track their proliferation index. T cells were then activated via the addition of CD3/CD28 Dynabeads and left to expand for 3 days (details in **Supplementary Materials**). Although the patient CD4+ cells did show proliferation, as evidenced by the decrease in overall CFSE signal intensity, it was significantly less than a panel of four healthy controls (**Figure 2A**). We note however that the dilution of CFSE over time is more noticeable in our patient cells than what has been described elsewhere (7), indicating a more robust response.

Additionally, as the literature indicates that STK4-deficient T cells show an exhausted phenotype prematurely (14), we also investigated this in our patient. T cells were stimulated for 2 days before assessing the helper T cell fraction for expression of PD-1, a surface marker for cellular exhaustion. Patient CD4+ cells did not show evidence of exhaustion (**Figure 2B**). This suggests that the increased PD-1 observed in other published STK4 patients may be a consequence of persistent EBV viremia, more than being an intrinsic part of the disease.

Loss of STK4 in both mice and humans is known to negatively impact T cell survival through increased apoptosis (6, 7, 15). We therefore evaluated activation-induced cell death (AICD) in the patient’s T cells by examining the effect of Fas binding on activated T lymphoblasts. PBMCs were activated for 48 hours with CD3/CD28 Dynabeads and then were mixed with or without anti-Fas antibody for an additional 24 hours before analysis of the DAPI/Annexin V apoptotic profile. In the absence of anti-Fas, the patient’s cells exhibited a greater propensity for apoptosis than controls (conducted only once due to cell limitations). In the presence of anti-Fas, both the control and the patient’s CD3^+^ cells displayed increased apoptosis, with the patient’s cells being significantly more susceptible to AICD (**Figures 2C, D**).

### Immunophenotyping reveals widespread dysregulation

**Figure 3:**
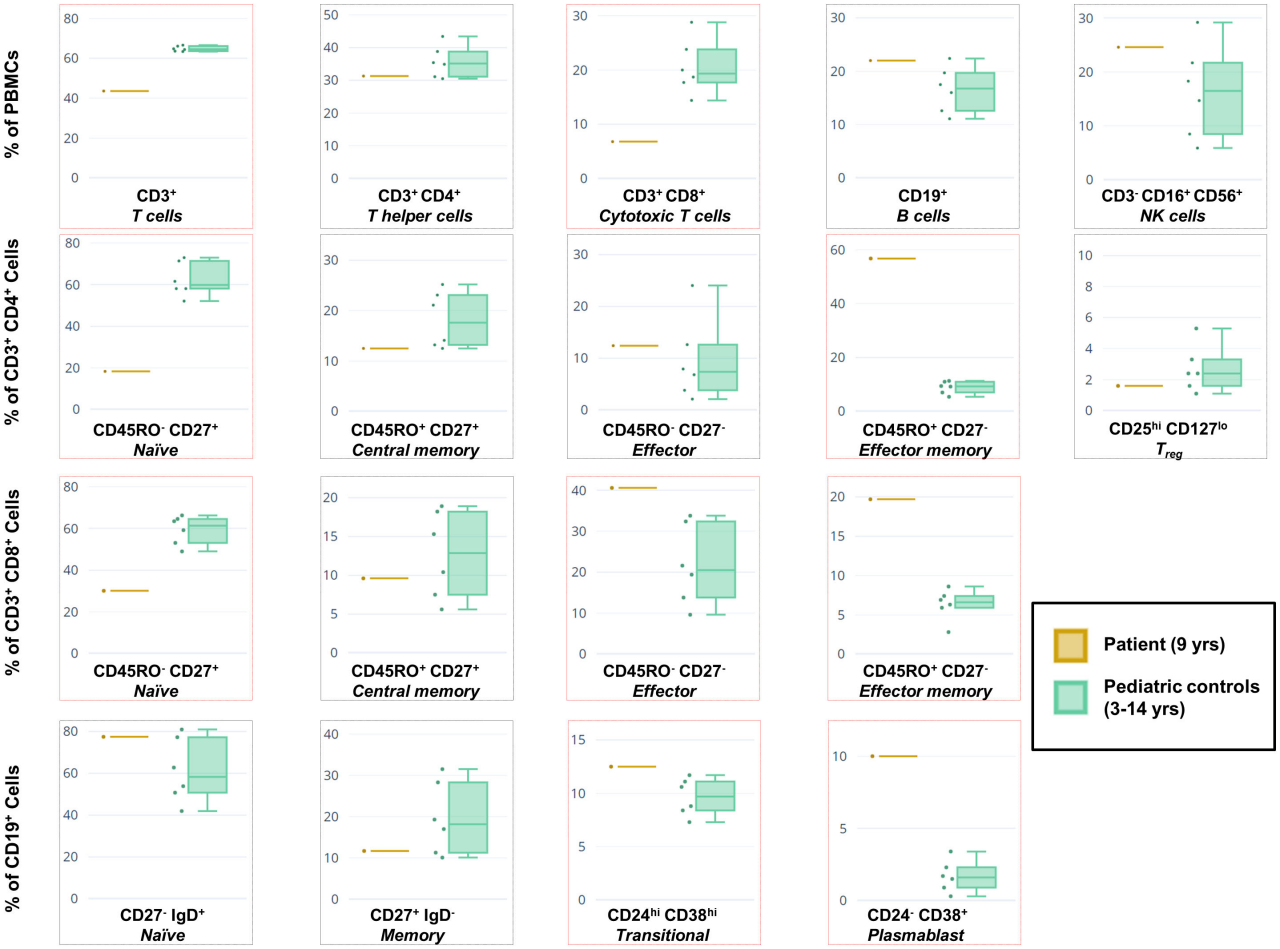
Immunophenotyping of the patient’s PBMCs versus a cohort of healthy pediatric controls (n = 6) using flow cytometry. Box-and-whiskers indicate major lymphocyte populations as well as CD4^+^ and CD8^+^ T cell, and CD19^+^ B cell compartments. Subpopulations for which our patient’s percentage is at variance with the combined data of the controls are boxed in red. Variance in data between this figure and **Table 1** (sampled roughly 1 year apart) is likely due to different sampling dates and changes in therapy, as well as the use of different protocols, antibody clones, fluorescent color combinations, and flow cytometers inherent to the clinical service versus the research laboratory.

To functionally assess the immunological effects of this mutation on our patient, PBMCs from the patient and age-matched healthy controls were stained with a panel of fluorescent antibodies prior to assessment using flow cytometry. This enabled us to evaluate the major lymphocyte populations, as well as specific T and B cell compartments (**Figure 3**). FACS analysis revealed widespread dysregulation of various populations, including strong decreases in the percentages of CD3^+^ T cells, CD8^+^ cytotoxic T cells, and naïve T cell compartments subtypes. Conversely, the effector memory subtypes of both CD4^+^ and CD8^+^ T cells were drastically overrepresented. Amongst B cells there was a trend of a high percentage of naïve cells and a low percentage of memory cells. The plasmablast subpopulation was substantially amplified as reported in other patients (16), although, unlike the overwhelming majority of cases, our patient exhibited normal IgG but high IgM levels. No obvious changes were detected in the NK or T_reg_ percentages as compared to controls.

## Discussion

To the best of our knowledge, this is the first report to showcase functional work on STK4-deficient human cells that still contain residual STK4 protein (i.e. are not a null phenotype). Previous mutations largely involved truncating or splice site changes that led to mRNA degradation and loss of detectable protein (6, 7, 11, 14, 17).

Our patient’s mutation is unique in that it removes the C-terminal SARAH domain only, and is present in the last exon which makes it resistant to NMD surveillance. In fact, while the amount of STK4 mRNA that is generated is significantly higher than in controls, the level of detected protein is significantly lower. The SARAH domain functions as a hydrophobic platform upon which proteins are able to form both hetero- and homodimers in anti-parallel orientation (13). Homodimerization, in particular, is crucial for allowing STK4 proteins to autophosphorylate each other, leading to their nuclear translocation and subsequent induction of cellular apoptosis via DNA fragmentation. The overwhelming majority of naturally occurring STK4 is found to be homodimerized, indicating that the monomeric form is not favored (18). We theorize that in our patient’s cells, loss of the SARAH domain and the consequent inability to undergo efficient autophosphorylation may have led to decreased stability and hence the reduced protein levels we observed.

STK4 activity can be modulated through the phosphorylation of certain residues contained within the SARAH domain (13). Interestingly, our patient’s mutation directly abolishes one of these, Ser438, which is an mTORC2 target that serves to inhibit STK4 and thereby facilitate cell proliferation. Phosphorylation of the nearby Thr440 triggers the opposite function by increasing STK4 activity (19, 20). It is intriguing to wonder if (and how) protein behavior might have changed had the frameshift mutation been located slightly further downstream, leaving one or both of these residues intact.

At both the clinical and cellular levels, there are remarkable differences between our patient and the consensus outlook reported in the literature. For example, STK4 deficiency is often associated with reduced IgM while our patient exhibits hyper IgM, a finding that has only ever been documented in one other case (17). Although our patient’s T cells are deficient in proliferation against a cohort of controls, following stimulation, they show similar speed to some slower controls (**Figure 2A**), unlike other patients in which the divergence is much more pronounced (7). Likewise, our patient’s T cells showed increased levels of apoptosis following continued stimulation (especially when challenged with anti-Fas, **Figure 2D**), but the percentage of apoptotic cells is not as high as cited elsewhere (6, 7).

It is interesting to note the duality of roles taken up by STK4 in immune cells. Aside from its function as tumor suppressor, it plays an anti-apoptotic role in hematopoietic cell lineages, protecting T cells from undergoing excessive apoptosis in knockout mice (15). Similarly, in humans, the null condition leads to dysregulation of immunophenotype and suppressed lymphocyte counts. Although this was noted in our patient, the extent was less severe than other reports. For example, the central memory compartments of both CD4^+^ and CD8^+^ T cell subpopulations, are not outside the bounds of what we observed in a healthy pediatric population, unlike the consensus of STK4 patients (16).

Patients with STK4 deficiency exhibit variable laboratory and clinical characteristics, and in accordance with previously published cases our patient demonstrated some of these common features such as intermittent neutropenia, and recurrent sinopulmonary infections. The latter, however, was controlled with immunoglobulin replacement therapy and prophylactic antibiotics. CD4 lymphopenia, the single most consistent finding in the literature, was indeed detected (**Table 1**) but not always consistently (**Figure 3**). Moreover, she was negative for other established features such as autoimmune disease, repeated skin viral infections, atopy, and EBV-associated lymphoproliferative disease. We hypothesize that this milder clinical phenotype, going hand-in-hand with the milder cellular phenotype, reflects the lack of NMD coupled with the presence of a low basal level of phosphorylation (in the absence of a SARAH domain that would facilitate full autophosphorylation). This leads to a partially rescued phenotype as compared to “null” cases.

Some of the previously reported patients exhibited congenital heart defects, however our patient’s echocardiography revealed no cardiac anomalies. In a recent cohort, all patients were evaluated by echocardiography regardless of symptoms and none were found to have heart defects. The authors concluded that the previously noted presence of cardiac abnormalities in STK4 patients may be coincidental (16).

A review of the literature reveals that 10 out of 26 reported patients underwent allogeneic hematopoietic stem cell transplantation (HSCT) (16, 21), with a post-transplant survival rate of 50%. Of these 10 patients, at least 7 were referred for transplant primarily due to EBV viremia/lymphoma. Our patient had no full-matched related stem cell donor, and a further search for unrelated stem cell sources was not done given the milder disease course.

Our data indicate that the presence of the STK4 peptide, in a truncated form that is missing the C-terminal SARAH domain, leads to largely improved clinical and laboratory findings as compared to the usually-reported “null” phenotype. Further work will need to be done to determine whether some patients, after considering the nature and location of their mutation, might benefit from future therapies that selectively modulate NMD activity. Such therapies may allow for the expression of truncated, partially-functional STK4 peptides with a stabilizing effect on lymphocyte survival and overall health.

## Data availability statement

The datasets presented here are protected under local data privacy laws, and cannot be made publicly available. Requests to access the datasets should be directed to the corresponding author (amalazami@kfshrc.edu.sa).

## Ethics statement

The King Faisal Specialist Hospital & Research Centre (KFSHRC) institutional review board approved all patient-related research, RAC # 2080 025. Informed consent was acquired from the parents for themselves as well as for the patient (as her legal guardians). Written informed consent was obtained from the participant/patient(s) for the publication of this case report.

## Author contributions

BA: Data curation, Supervision, Writing – review & editing. HuA: Data curation, Investigation, Methodology, Writing – review & editing. HiA: Data curation, Investigation, Methodology, Writing – review & editing. LA: Data curation, Writing – review & editing. AA: Data curation, Writing – review & editing. HG: Data curation, Investigation, Writing – review & editing. MA: Data curation, Formal analysis, Investigation, Supervision, Writing – review & editing. AMA: Formal analysis, Methodology, Project administration, Supervision, Writing – original draft.
