# Supplementary material for: A unique STK4 mutation truncating only the C-terminal SARAH domain results in a mild clinical phenotype despite severe T cell lymphopenia: Case report
Source: Front Immunol. 2024 Feb 1;15:1329610. doi: 10.3389/fimmu.2024.1329610 (PMC10867200; doi:10.3389/fimmu.2024.1329610)
Supplement: Supplementary file 1 [file DataSheet_1.pdf]

# A Unique STK4 Mutation Truncating Only the C-Terminal SARAH Domain Results in a Mild Clinical Phenotype Despite Severe T Cell Lymphopenia: Case Report

## SUPPLEMENTARY MATERIALS

### Study Approval

The King Faisal Specialist Hospital & Research Centre (KFSHRC) institutional review board approved all patient-related research, RAC # 2080 025. Informed consent was acquired from the parents for themselves as well as for the patient (as her legal guardians). Clinical and family histories were documented at the time of blood sampling. PBMCs were isolated using Ficoll density gradient centrifugation for cryopreservation.

### Next-Generation Sequencing (NGS)

Blood-derived genomic DNA was subjected to whole exome sequencing (WES) utilizing SureSelectXT Target Enrichment (Illumina NovaSeq 6000, San Diego, CA, USA). Raw data quality metrics were calculated using FastQC, and the reads were aligned to the human reference genome (hg19) using Burrows-Wheeler Aligner (BWA)21. The resultant bam files were evaluated for PCR duplicates. Picard (http://broadinstitute.github.io/picard) and GATK22 were used to execute local realignment and base-quality recalibration, respectively, to ensure high-quality base calls. MuTect223 was utilized for identifying somatic mutations, and ANNOVARv21.07 for annotating mutations with various databases. Single nucleotide variants (SNVs) and indels that passed the standard MuTect2 filters underwent additional filtering as indicated in Fig 1A.

## Sanger Sequencing

Genomic DNA was acquired from whole blood samples. Primers containing 5’-tagged M13 sequences were designed as required. DNA sequencing was conducted using the BigDye Terminator (Thermo Fisher, Waltham, MA, USA). The Saudi Human Genome Database was queried to determine whether variants highlighted by WES were unreported SNPs segregating in the general population.

## RNA Expression Analysis

Lymphoblastoid cell lines were generated via standard protocols from the patient and three healthy controls. Following RNA extraction and cDNA synthesis, real-time reverse-transcriptase PCR (Applied Biosystems 7500 Fast Real-Time PCR System, Thermo Fisher) was conducted with exon–exon spanning primers. Primer sequences were 5′-CAATCAACTCTTAAGGGACTTGAA -3′ and 5′- CTGAATCACTTCTGGAGCCAT-3′. Quantification was performed using the QuantiTect SYBR Green mix (Qiagen, Limburg, Netherlands), and the deltaCt technique was employed to quantify fold difference. Reactions were conducted in triplicate each time, with at least two independent runs, then normalized to GAPDH as the internal control.

Immunoblotting

Lymphoblastoid-derived cell lysates were prepared with RIPA buffer (Sigma, St. Louis, MO, USA) and quantified on a Smart-Spec Plus spectrophotometer (Bio-Rad, Hercules, CA, USA) using the Protein Assay Dye Reagent (Bio-Rad). Protein samples were electrophoresed on an SDS-PAGE (National Diagnostics, Atlanta, GA, USA), and transferred onto a PVDF membrane (GE Healthcare, Waukesha, WI, USA). Primary antibodies utilized in this study included: anti-GAPDH (#2118), anti-MST1/STK4 (#3682) and anti-FoxO3a (#2497) (Cell Signaling Technology, Danvers, MA, USA).

Immunophenotyping

Frozen PBMCs were thawed and rested for 2 hours in complete medium, then counted and suspended at $10^6$ cells per ml in FACS buffer (2% FBS in PBS) before being stained and analyzed on an LSR II flow cytometer (Becton Dickinson, Mountain View, CA). All antibodies were acquired from BD Biosciences (Franklin Lakes, NJ, USA). T cell analysis utilized antibodies against CD3-Amcyan, CD4-PerCP-Cy5.5, CD8-PE-Cy7, CD45RO-FITC and CD27-PE. Naïve ($CD45RO^-CD27^+$), central memory ($CD45RO^+CD27^+$), effector memory ($CD45RO^+CD27^-$) and effector ($CD45RO^-CD27^-$) T cell subsets from gated from $CD3^+/CD4^+$ or $CD3^+/CD8^+$ cells. Regulatory T cells ($T_{reg}$) were classified as the ($CD25^{hi}/CD127^{lo}$) subset of $CD3^+/CD4^+$ cells. NK cells ($CD3^-/CD16^+/CD56^+$) were identified using antibodies against CD3-Amcyan, CD16-APC and CD56-PE. For B cells, antibodies against CD19-PerCP-Cy5.5, CD27PE-Cy7, CD38-APC, CD24-PE, IgD-FITC and IgM-APC-Cy7 were used to designate the naïve ($CD27^-IgD^+$), memory ($CD27^+IgD^-IgM^-$), transitional

($CD24^{high}CD38^{high}$) and plasmablast ($CD24^{-}CD38^{+}$) fractions from gated live ($DAPI^{-}$) $CD19^{+}$ cells.

Flow Cytometry

For the carboxyfluorescein 6 succinimidyl ester (CFSE) proliferation assay, thawed PBMCs were rested for 2 hours in RPMI 1640 containing 10% fetal calf serum (FCS) and 1% penicillin/streptomycin (all from Gibco, Billings, MT, USA). Cells were then stained with 1 µM CFSE (BD) at 37 degrees for 15 minutes, washed extensively in complete medium, then seeded in a U-bottom 96-well plate at a density of 1 x $10^6$ cells/ml. PBMCs were incubated in the presence or absence of Dynabeads Human T-Activator CD3/CD28 (Thermo Fisher). After 72 hours cells were stained with anti-CD4-APC (BD), along with 4′,6-diamidino-2-phenylindole (DAPI) for live/dead screening, and analyzed on an LSR II flow cytometer.

For programmed cell death protein 1 (PD-1) cell surface detection, thawed PBMCs were rested as above then stained with anti-CD4-APC, anti-PD-1 (eBioscience, San Diego, CA, USA) and DAPI, prior to running on flow cytometry (day 0 analysis). Day 2 analysis was conducted following a 48 hour incubation of the cells with CD3/CD28 Dynabeads.

To assess activation-induced cell death (AICD) of T cells, PBMCs were incubated with Dynabeads for 48 hours. Activating anti-Fas antibody (Sigma 05-201) was then added at a concentration of 1 µg/ml, and cells were incubated for an additional 24 hours prior to gating with anti-CD4-APC and analyzing DAPI vs Annexin V-Alexa Fluor 488 (Invitrogen V13241).
